# Japanese cancer patient participation in and satisfaction with treatment-related decision-making: A qualitative study

**DOI:** 10.1186/1471-2458-8-77

**Published:** 2008-02-27

**Authors:** Yoshiko Watanabe, Miyako Takahashi, Ichiro Kai

**Affiliations:** 1 Department of Social Gerontology, School of Public Health, University of Tokyo, Tokyo, Japan

## Abstract

**Background:**

Over the last decade, patient involvement in treatment-related decision-making has been widely advocated in Japan, where patient-physician encounters are still under the influence of the long-standing tradition of paternalism. Despite this profound change in clinical practice, studies investigating the actual preferences of Japanese people regarding involvement in treatment-related decision-making are limited. The main objectives of this study were to (1) reveal the actual level of involvement of Japanese cancer patients in the treatment-related decision-making and their overall satisfaction with the decision-making process, and (2) consider the practical implications of increased satisfaction in cancer patients with regard to the decision-making process.

**Methods:**

We conducted semi-structured interviews with 24 Japanese cancer patients who were recruited from a cancer self-help group in Tokyo. The interviews were qualitatively analysed using the approach described by Lofland and Lofland.

**Results:**

The analyses of the patients' interviews focused on 2 aspects: (1) who made treatment-related decisions (the physician or the patient), and (2) the informants' overall satisfaction with the decision-making process. The analyses revealed the following 5 categories of decision-making: 'patient as the active decision maker', 'doctor selection', 'wilfully entrusting the physician', 'compelled decision-making', and 'surrendering decision-making'. While the informants under the first 3 categories were fairly satisfied with the decision-making process, those under the latter 2 were extremely dissatisfied. Informants' views regarding their preferred role in the decision-making process varied substantially from complete physician control to complete patient control; the key factor for their satisfaction was the relation between their preferred involvement in decision-making and their actual level of involvement, irrespective of who the decision maker was.

**Conclusion:**

In order to increase patient satisfaction with regard to the treatment-related decision-making process, healthcare professionals in Japan must assess individual patient preferences and provide healthcare accordingly. Moreover, a better environment should be created in hospitals and in society to facilitate patients in expressing their preferences and appropriate resources need to be made available to facilitate their decision-making process.

## Background

Over the last two decades, patient participation in treatment-related decision-making has been promoted as being ethically and clinically desirable in Western countries [1-3]. Several studies have indicated that patient participation in decision-making has a positive influence on their health outcomes, thereby increasing patient satisfaction regarding medical care and promoting patient autonomy [4-6].

Charles and colleagues provided useful suggestions for developing a framework for the analysis of treatment-related decision-making and proposed 3 analytical approaches that have been reported in the recent history of developed countries: the paternalistic approach, characterised by physician control; the informed approach, characterised by division of labour and preservation of patient autonomy; and the shared approach, characterised by simultaneous interaction between both the patient and physician in all stages of the decision-making process [7]. Further, they suggested that decision-making is not merely the act of 'making a decision'; in fact, it is an interactive process between a patient and her/his physician. The process consists of 3 analytically distinct steps based on which the 3 approaches (paternalistic, informed, and shared) can be compared and differentiated. The 3 steps are (1) exchange of information, (2) deliberation regarding treatment options, and (3) decision-making related to the treatment to be implemented [8]. They argue that the third step is an outcome of the deliberation process.

Despite the fact that patient involvement in treatment-related decision-making has been widely advocated and promoted in both clinical and policy-making settings in many developed countries, research conducted in the US, Canada, and the UK revealed that people's preferences regarding their role in the decision-making process vary substantially [9-15]. Some research further indicated that the preference for handing over the control to the physician is significantly greater when the situation involves potential mortality or when the respondents' health status is deteriorating [9-11,15]. Moreover, other studies have revealed that the relationship between patient preferences regarding their involvement in the decision-making process and their actual level of involvement is a strong indicator of patient satisfaction [12,14]. Thus, it has been increasingly emphasised that (1) healthcare professionals need to assess individual patient preferences in order to provide tailor-made care accordingly [14,15] and (2) merely pressurising patients to decide a treatment option could have negative psychosocial consequences, if the patient does not wish to be the final decision maker [14].

Japan has had a long-standing tradition of paternalism with regard to patient-physician encounter. Nomura and colleagues [16] described the dominant category regarding the patient-physician relationship in Japan as follows: 'the relationship between a [Japanese] physician and a patient is clearly asymmetrical, since the patient seeks help and care from a medical expert whose diagnostic evaluations are more or less indisputable and whose decisions have to be accepted by the patient without discussion'. This type of a traditional decision-making process in which the patient leaves the decision to her/his physician has been described by researchers and termed in Japanese as the '*Omakase *(entrusting)' style [17,18]. The *Omakase *style of decision-making can be encountered in many scenarios of everyday life in Japan where the experience, knowledge, and advice of experts is highly respected. However, the need to recast the traditional paternalistic patient-physician relationship has become sufficiently pervasive, and the relationship is, therefore, undergoing a gradual transformation [19,20]. In 2004, Japan Medical Association (JMA), the largest medical professional body in Japan, issued the Professional Ethics Guideline for Physicians [21] that provides a detailed explanation of the professional code issued by the JMA in 2000 and stipulates that physicians have an ethical obligation to disclose in entirety all relevant information to the patient in a comprehensible manner. Although the Guideline also provides physicians the freedom to withhold information if deemed 'appropriate' [22], it declares the basic standpoint of the JMA which is to strongly endorse the principle of information disclosure and obtain informed consent from patients.

Over the years, clinical practice in Japan has witnessed considerable changes with regard to the patient-physician relationship. Although respecting patient autonomy is regarded as important from various aspects of a patient-physician encounter, there is a paucity of studies investigating the actual preferences regarding the involvement of Japanese people, who have been under the influence of the *Omakase *culture for a long time, in treatment-related decision-making. Some studies suggest that the preferences of Japanese patients, as well, regarding participation in the decision-making process vary substantially. Research involving Japanese hypertensive outpatients using the Autonomy Preference Index has suggested that patients had an intermediate desire to be involved in decision-making and a greater desire for information [16]. Another research on diabetic outpatients randomly assigned 1 out of 3 case study vignettes (pneumonia, gangrene, or cancer) to patients and enquired about their attitudes towards participation in the decision-making process [23]. The overall percentage of respondents who preferred active, collaborative, and passive roles was 12%, 71%, and 17%, respectively, respondents to the cancer vignette being less likely to prefer an active role as compared to the non-cancer vignette respondents. These studies, however, investigated patient preferences under hypothetical situations and did not include patient satisfaction as an outcome.

Research investigating patient preferences in the decision-making process in the context of cancer is even scarcer. It deserves more attention because cancer affects a majority of Japanese people, being the primary cause of death [24] and the estimated lifetime prevalence being as high as 46.3% for men and 34.8% for women [25]. Further, under the trend of information disclosure and promoting informed consent in Japan over the last decade, Japanese physicians have drastically shifted their attitudes towards disclosing cancer diagnosis, which, earlier, used to be undisclosed to patients [26-28]. For example, one research that reviewed the medical and nursing charts of a hospital suggested that the percentage of patients to whom cancer diagnosis was disclosed increased from 27% in 1993 to 71% in 1998 [28]. Japanese physicians should now begin discussing treatment options, including the risks and benefits, with their patients in an open awareness context [29]; however, not all physicians are well equipped for this since they lack adequate communication skills.

Given the above-mentioned backdrop, we decided to investigate the current situation of treatment-related decision-making in the oncology setting in Japan. In this preliminary research, we hoped to enquire from Japanese cancer patients about their own experiences, not hypothetical situations, regarding the decision-making process. The main objectives of this study were to (1) reveal the actual level of involvement of Japanese cancer patients in treatment-related decision making and their overall satisfaction with the decision-making process, and (2) consider its practical implications in order to increase the cancer patients' satisfaction with the decision-making process.

## Methods

Due to the paucity of researches on treatment-related decision-making in Japanese cancer patients, we decided to conduct qualitative, semi-structured interviews in order to obtain detailed descriptions of the variations in the experiences of the informants with regard to their participation in the decision-making process.

### Recruitment of interview respondents

The informants were recruited from a cancer self-help group in Tokyo. The group, which was established 20 years ago by a man with hepatoma, consists of 430 cancer survivors who reside in the Tokyo metropolitan area. The group does not limit itself to members of a particular age, gender, or cancer site and operates on a membership fee of ¥5000 (approximately US$47.00) per year. Occasionally, healthcare providers participate as supporters, providing medical information; however, they do not take any initiative in the administration of the group. We decided to contact this particular group because we wished to maximise the informants' demographic and clinical backgrounds in order to explore a wide range of patient-physician encounters in the context of treatment-related decision-making among cancer patients in Japan. Moreover, the fact that all the members of this group were well informed about their cancer diagnosis made it optimal for exploring their experiences in an open awareness context.

Two authors (YW and MT) attended the monthly meetings of the self-help group in 2001 and explained verbally as well as in the form of flyers the purpose and procedures of the research and its ethical considerations. Further, we emphasised that we would prefer to recruit informants from various backgrounds. Regarding the ethical considerations, the ethical regulations in Japan as of 2001 did not require this study to be submitted for scrutiny by the ethical review committee. Nevertheless, we employed a number of ethical considerations while undertaking the research, including voluntary participation of the respondents, their right to withdraw from the study at any point of time, and the assurance that the data would not be used for other purposes and all information would be kept strictly confidential, and explained them to the participants. Participants who chose to be involved in the research were instructed to contact the principal author, and a convenient time and place to conduct the interview was negotiated.

### Data collection

After obtaining informed consent, semi-structured interviews were conducted by the principal author, who is a registered nurse with clinical experience in the oncology setting. Interviews were conducted at a place chosen by the respondents themselves, in order to protect their privacy and create a comfortable atmosphere for discussion; these places included informants' homes, community centres, coffee shops, and hospital waiting areas. The informants were awarded a book coupon worth ¥1000 (approximately US$9.00) for their participation. With the participants' permission, all interviews except one were tape-recorded. For the informant who declined the use of a tape recorder, the interviewer made notes by writing down the informant's responses as accurately as possible; these notes were used as supplementary data for the analysis. On average, the interviews lasted for a duration of 80 minutes, ranging between 55 and 200 minutes, and a total of 35 hours of interview data was collected.

The interviews began with questions regarding the informant's socio-demographic and clinical backgrounds followed by the treatment-related information provided by her/his physician, the process of interaction with the physician until the treatment plan was decided, and the overall satisfaction with the treatment-related decision-making process. Further, we enquired about the informants' experiences in decision-making regarding the critical treatment modalities that might have considerably affected the course of their disease, such as surgeries, radiation therapy, and chemotherapy. As the interview data accumulated, we attempted to include more questions in order to delineate the similarities and differences among informants who adopted different styles of participation in the decision-making process. For example, to compare the informants who attempted to actively make decisions with those who left the decisions to the physicians, we enquired about the reasons why they did so as well as the factors that they considered important in making that particular decision. Immediately after each interview, the interviewer (YW) wrote field notes regarding the impression of the interview.

Apart from conducting interviews, the principal author participated in the regular monthly meetings of the group over a period of 10 months in order to acquire an understanding of the background issues experienced by the group members. The field notes obtained during the observation were used as supplementary data while analysing the interviews.

### Data analysis

Data analysis was conducted concurrently with the interview process according to the procedures described by Lofland and Lofland [30]. All the interviews that were recorded on tape were transcribed verbatim. Subsequently, we repeatedly read the transcripts line by line and placed conceptual labels accordingly. Many informants experienced more than one opportunity to make a decision, and we assumed that the patients' decision-making style might have varied under different circumstances; therefore, the unit of the analysis was each opportunity to make a decision rather than the informant herself/himself. Multiple conceptual labels were compared according to their similarities and differences and were grouped together to form loosely defined categories. Thereafter, the preliminary categories created by the principal author were scrutinised by the other authors to verify whether the categories appropriately explained the variations in the decision-making styles. MT is a physician researcher with clinical experience in the oncology setting and expertise in qualitative research methodology. IK is also a physician researcher who is well versed with quality-of-life issues and patient-physician relationships in Japan. First, we categorized each decision-making opportunity according to the level of patient involvement, which was based on who was the final decision maker in each decision-making opportunity. However, we soon realised that the level of patient satisfaction with the overall decision-making process was not necessarily related to who made the final decision – the patient or the physician. Therefore, we compared each opportunity of decision-making based on 2 criteria: the final decision maker and the informants' overall satisfaction. To ensure the validity of the analysis, we sent its final version to 2 of the informants (informant D and Q) for the purpose of member checking [31]. These 2 informants were selected because they were the core members of the self-help group and could be consulted by other group members regarding problems in patient-physician encounters. Based on their feedback, it can be stated that the authors' interpretations adequately explained the informants' experiences.

## Results

A total of 24 interviews were conducted with 10 men and 14 women. The background information of the informants is presented in Table 1. The mean age of the informants was 57.8 years (range, 36–78 years) and the median duration from diagnosis to interview was 5 years (range, 6 months-17 years). The primary cancer sites were the lungs, esophagus, breasts, pancreas, liver, stomach, uterus, colon, prostate, cartilaginous tissue, and lymph nodes. Of the total informants, 2 had cancer involving multiple sites, and 6 had cancer recurrence.

**Table 1:** Socio-demographic backgrounds of the informants

Informant	Gender	Age	Education	Cancer type (primary)	Time elapsed since diagnosis
A	Female	57	Junior College	Breast Cancer	5 yrs 3 mths
B	Male	53	Graduate School	Colon Cancer	10 mths
C	Female	50	Junior College	Lung Cancer	6 mths
D	Female	58	High School	Multiple Cancer ^a^	^a^
E	Male	65	University	Multiple Cancer ^b^	^b^
F	Female	53	High School	Colon Cancer	4 yrs
G	Male	59	Unknown	Liver Cancer	10 mths
H	Male	66	University	Lung Cancer	2 yrs 7 mths
I	Male	53	University	Lung Cancer	9 yrs 2 mths
J	Male	60	University	Colon Cancer	5 yrs 1 mth
K	Female	61	High School	Breast Cancer	8 yrs 5 mths
L	Female	64	Career College	Malignant Lymphoma	12 yrs
M	Female	62	High School	Breast Cancer	8 yrs
N	Female	39	Graduate School	Colon Cancer	1 yr 9 mths
O	Male	59	University	Pancreatic Cancer	5 yrs
P	Female	42	University	Gastric Cancer	1 yr 3 mths
Q	Male	74	University	Colon Cancer	16 yrs
R	Male	58	University	Esophageal Cancer	4 yrs
S	Female	53	University	Breast Cancer	4 yrs 9 mths
T	Female	70	High School	Uterine Cervical Cancer	13 yrs
U	Female	60	High School	Breast Cancer	9 yrs 6 mths
V	Male	78	University	Prostate Cancer	3 yrs 8 mths
W	Female	57	High School	Colon Cancer	3 yrs 1 mth
X	Female	36	Unknown	Colon Cancer	2 yrs 6 mths

^a ^Gastric cancer (17 yrs), colon cancer (7 yrs 6 mths), chondrosarcoma (6 yrs)

^b ^Colon cancer (14 yrs), lung cancer (7 yrs)

**Figure 1:**
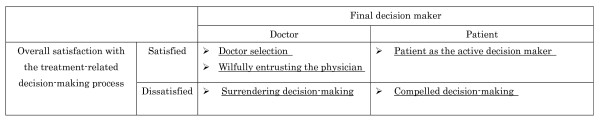
Patient participation in and overall satisfaction with the decision-making process.

During the course of the analysis that focused on the final decision maker and the informants' overall satisfaction, the following 5 categories of decision-making emerged: patient as the active decision maker, doctor selection, wilfully entrusting the physician, compelled decision-making, and surrendering decision making (Figure 1). A detailed description of each category has been provided below.

### Patient as the active decision maker

In this category, informants described themselves as active participants in the decision-making process and recalled the process with a high level of satisfaction. The informants in this category had their own opinions, beliefs, and values with regard to their treatment.

*I have my own philosophy that physicians simply treat diseases, and merely following their advice does not necessarily save patients' lives. I know many people who have not survived even after following everything recommended by their physicians. Why don't patients participate in their own treatment-related decision-making? They should. I believed that the only way to survive was to be involved in the decision-making process regarding my treatment and not leaving the decision in the hands of the physician. ...Although my physician advised me to take an oral anti-cancer drug, I refused to do so because I had never come across any data suggesting that the drug was effective in treating the cancer that I had.* (Informant O)

Informant O expressed a strong belief in his own self-determination and assertively appealed to his physician to respect his preference, thus rejecting the oral chemotherapeutic drugs that the physician had advised. In this category, physicians are regarded as consultants with special knowledge and skills that patients may utilize as important sources of information. The decision-making process adopted by Informant O can be described as an 'informed approach', as proposed by Charles and colleagues, which is characterised by the division of labour and preservation of patient autonomy [7,8].

It is of interest that the informants in this category often stated that they had gradually adopted this decision-making style after experiencing confusion in the initial treatment phase when they were forced to make decisions without adequate knowledge, as in the case of Informant I.

*At the first hospital, nobody informed me about the nature of my disease. Fortunately, at the next hospital, the physician provided me with all the necessary details regarding my disease; however, he compelled me to make crucial decisions that would ultimately kill or cure, despite my extremely limited knowledge about the disease. I experienced myriad problems and consequently changed the manner in which I participated in the decision-making process. A patient must, all by himself, determine whether the physician is correct or not. For this reason, I visited another hospital for a second opinion. If you want to make your own choice, you must have the necessary knowledge. Therefore, I investigated, sought information, and asked questions. I challenged myself to listen to people who had different ideas with regard to my treatment and read the books that were written by them. Subsequently, I chose the physician who offered convincing answers to my questions. I refer to this as participation in a decision-making process. ...Now, I strongly believe that a patient and a physician should, as equal partners, discuss the best treatment modality for the patient and make a combined decision. Hence, I now adopt this style of decision-making.* (Informant I)

Informant I stated that he felt as though his physician had shifted the responsibility of decision-making onto him, despite his unpreparedness. As a result, he became more assertive and approached his physician for explanations regarding his disease and treatment options; in addition, he gathered information from other resources to better understand the physician's explanations. We can associate Informant I's style to the 'shared approach' described by Charles and colleagues, which is characterised by the discussion of treatment options by both the patient and physician as equal partners and the sharing of the responsibility for decision-making [7,8].

Interestingly, informants who indicated that they played an active role in the decision-making process did not necessarily confirm whether, from their perspective, they were the final decision makers (Informant O) or they shared the responsibility of decision-making with their physicians (Informant I). It is probable that informants did not need to distinguish between the above 2 possibilities because in either case, the patients had discussed their treatment-related decisions with their physicians and had expressed their opinions assertively, thus challenging the long-standing norm of *Omakase *in Japan.

### Doctor selection

In the second category, the informants were eager to gather information on the quality of hospitals and physicians and wished to be informed as much as possible about their treatment options; however, they still intentionally left the final decision to the physician. Since the act of decision-making was transferred to the physician, this category can be referred to as 'paternalistic', considering the issue of making decisions *per se*; however, the informants maintained a sense of self-determination and showed a high level of satisfaction.

*I attempted to gather as much information as possible on the reputation of various physicians and hospitals. Moreover, I went to visit different physicians in person. ...Patients' knowledge regarding medicine is miniscule as compared to that of the physicians, which is, probably, a hundred times greater. Given this information gap, it is futile for me to attempt to determine whether radiotherapy and chemotherapy or surgery is a better treatment option for me. Choosing the right physician and asking him to decide is crucial*. (Informant Q)

*We need to acquire accurate knowledge about our own medical status and choose the most suitable physician accordingly. I first asked my physician for his opinion and then gathered considerable information from the media, the Internet, etc. in order to find data that endorsed his opinion. Since physicians possess detailed knowledge of medicine and have considerable clinical experience, I was unsure whether it was really necessary for me to make the final decision. I trusted my physician. The ultimate purpose was to cure my disease and not to personally make the final decision. The fact that a treatment option is chosen by a patient does not necessarily guarantee its effectiveness. If one understands the treatment and agrees with it, it is irrelevant as to who makes the decision. Therefore, it is perhaps reasonable to say that 'active participation' can be rephrased as 'active agreement'.* (Informant B)

In this category, the patients collected information to find the right physician who would choose the appropriate treatment option for them or to endorse the physician's advice and understand it more thoroughly; however, it was not to empower themselves in order to make the right treatment-related decisions.

### Wilfully entrusting the physician

In the third category, the informants entirely believed in and trusted the physician's professionalism in general and did not feel the need to evaluate the quality of each physician or to understand the content of the advice provided. Therefore, they felt no desire to collect information regarding a specific physician's reputation or the specific treatments. In this category, the physician was the final decision maker for her/his patient's treatment, and the patient's role was to accept the physician's treatment decisions without question. This category can be regarded as Charles and colleagues' 'paternalistic approach', which is characterised by giving the physician complete control [7,8]. Given the current trend of patient-centred practice in Japan, despite the negative connotation of the word 'paternalism', the informants expected the physicians to make decisions for them and were quite satisfied with the overall decision-making process.

*Basically, I trust physicians. In general, physicians, like lawyers, have a strong philosophy of life, and therefore, I respect them. My physician only informed me that 'there would be no problems if you undergo surgery soon'. I had a strong feeling that he was reliable, maybe, in part, due to his personality. ...Since he said it would be fine, I did not discuss treatment options with him any further. I trusted him, and thereafter, I adopted the Omakase approach.* (Informant T)

### Compelled decision-making

In the fourth category, the informants felt that they were compelled to make a decision, even though they did not have sufficient information or understanding regarding their medical condition and treatment options. Although they opted for a particular treatment from the myriad options presented to them, they argued that the decision was a forced act and that the responsibility of making the decision was imposed on them by their physicians. The informants' recollection of the situation was considerably negative, and their overall satisfaction with the decision-making process was extremely low. Informant I, who was introduced in the 'patient as the active decision maker' category, stated that he was also compelled to make a decision during the initial treatment phase and recalled the interaction with his physician with a sense of bitterness.

*At the time of making the decision regarding which treatment to undertake, I had to choose one treatment option and provide my physician with an answer. This was not something that one could satisfactorily refer to as 'shared decision-making'. I felt that I had to reach a conclusion despite my lack of medical knowledge. Perhaps, by choosing one treatment option from the many options that were presented to me, I did take active part in the decision-making process; however, I do not consider it to be actual participation. It was merely the case of an ignorant person being forced to make a decision. *(Informant I)

In this case, Informant I stated that the problem was his lack of medical knowledge. If, however, he was provided with detailed information on his medical status and treatment options and was given sufficient time to deliberate, he might have been able to confidently decide his treatment. Moreover, interviews revealed another kind of compelled decision-making in which the patient was forced to make the final decision against her/his will to leave the decision to the physician. This is a typical example of the disparity between patient preferences in decision-making and their actual level of involvement.

*I did not want to decide on my own; however, he (the physician) informed me that I was the decision maker. This troubled me, and from then on, I could not sleep well. I wish the physician had made the decision for me. I wanted the physician to lead me in the right direction. I was completely confused because the physician had imposed the responsibility of decision-making on me.* (Informant R)

### Surrendering decision-making

In the last category, the informants surrendered the control of decision-making to the physician and recalled the entire process with a strong sense of resignation. This category reflects the long-standing tradition of physicians' domination in the decision-making process in Japan. Although the informants actually wished to play a more active role, they rejected the idea because they perceived a marked difference in authority between themselves and their physicians, and consequently, accepted the physician's advice without question. Therefore, when their physicians chose a certain treatment option for them without providing them with the necessary knowledge on other treatment options or the opportunity to express their preferences, they assumed that they had no other choice but to entrust their physicians to make the best decision for them. This category can also be described as the 'paternalistic approach'; however, unlike the 'wilfully entrusting the physician' category, the informants in this category displayed dissatisfaction and a deep sense of resignation with the overall decision-making process.

*I cannot say that I was satisfied with the decision-making process; however, I thought that I was not in a position to assert myself and had to accept my physician's decision. As a patient, I thought there was nothing I could offer, even though I wanted to participate in the treatment-related decision-making process. If the physician had provided me information about the available treatment options, I think I could have chosen one. The physician should have voluntarily explained to me what the best option was, providing reasons for choosing it and explaining the advantages and disadvantages of the other options.* (Informant K)

*Physicians often propose the idea of informed consent to indicate that they respect the patients' will; however, in fact, they are persuasive and assertive and enforce their opinions on their patients. That is the way physicians are, I think.* (Informant D)

Although informant D accuses physicians in general of imposing their opinions on the patients, she describes the situation as 'that is the way physicians are' and does not dare to inform her physician about her desire to participate more actively in the decision-making process.

## Discussion

This study aimed to explore the actual level of involvement of Japanese cancer patients in treatment-related decision-making as well as their overall satisfaction with the decision-making process. The strength of this study is in that we asked the patients about their actual experiences; therefore, we could not only explore the actual level of involvement of the patients in the decision-making process but also reveal the situations in which the patients were dissatisfied.

This study revealed that Japanese cancer patients' preferences regarding their role in treatment-related decision-making varied widely from complete physician control to complete patient control, which is similar to the preceding studies conducted in the US, the UK, and Canada [9-12,14,15]. Further, this study suggested that the correlation between patient preferences regarding their involvement in the decision-making process and their actual level of involvement was a strong indicator of patient satisfaction, which was independent of who made the final decision. Moreover, this finding is compatible with other studies conducted in the US [12,14]. Patient involvement in decision-making has been widely advocated in Japan, presumably as a reaction to the historical paternalistic patient-physician relationship [32]. However, it is preferable that physicians be aware that merely allowing the patients to decide their treatment option regardless of their preferences may cause confusion rather than satisfaction among patients. Instead, assessing each patient's preference regarding her/his role in the decision-making process is extremely important in order to provide customized, preference-sensitive care. It was also observed that some physicians forced their patients to determine their treatment without providing them with sufficient treatment-related information. It appears that these physicians attempted to involve the patients only in decision-making and not in the preceding important steps of information exchange and deliberation [8]. Therefore, it is preferable for physicians to be informed that treatment-related decision-making is the outcome of information exchange and deliberation regarding the treatment options, as proposed by Charles and colleagues [8].

Another interesting finding of this study is the 3 distinct categories in which the patients allowed the physician to make the final decision: 'wilfully entrusting the physician', 'surrendering decision-making', and 'doctor selection'. 'Wilfully entrusting the physician' and 'doctor selection' can be regarded as the 'active *Omakase*' model, and 'surrendering decision-making' can be considered to be the 'passive *Omakase*' model. These models have been described in detail by Slingsby, who conducted interviews with Japanese psychiatrists and analysed, from the psychiatrists' perspective, the patient-physician relationship in the treatment of minor mood disorders in Japan [18]. It would be reasonable to state that from the perspective of the cancer patients, the 'wilfully entrusting the physician' and 'surrendering decision-making' categories in this study endorse the presence of 2 kinds of *Omakase *styles. The third category, 'doctor selection', is also of interest. This category is different from the previous 2 categories in that the patients are eager to gather information on the quality and reputation of hospitals and physicians and wish to be informed as much as possible regarding treatment options. This may be a category characteristic to a country like Japan where all people are covered by the National Health Insurance System and are allowed to choose any physician of their choice [33]. In such a healthcare system, patient participation begins at the stage when the patient chooses a physician for herself/himself, and hence, patients can maintain a sense of autonomy and self-determination even if they do not decide their treatments themselves. Hashimoto and Fukuhara investigated the Japanese general population and reported that people's preferences with respect to acquiring information was not associated with their preference regarding their role in the decision-making process, particularly among individuals who believed that their health was dependent on other influences such as physicians, family, and friends [34]. They speculated that such individuals may use the information for purposes other than rational decision-making, such as anticipating what is going to happen next. The 'doctor selection' category is an example of the manner in which information is used by patients for purposes other than rational decision-making.

This study has several limitations which should also be acknowledged. The first is the recall bias which was a result of the interviews being conducted retrospectively; thus, the findings might have been influenced by a number of factors, including the time elapsed since the patient underwent treatment and the patients' current health status. Second, the fact that most of the informants of this study reside in the Tokyo metropolitan area reduces the generalizability of the findings of this study. We can speculate that if the patients had been residing in a rural area, where the access to both medical facilities and healthcare information is limited and the patient-physician relationship is under the influence of more conservative socio-cultural norms, their decision-making categories might have differed from those described in this study. Third, all informants were not in the terminal stage of the disease at the time of the diagnosis and thus, had a high chance of survival for at least a certain period of time. If the respondents were in the advanced stage of cancer at the time of diagnosis, it might have influenced their preference regarding their role in the decision-making process. Furthermore, during the interviews, we did not necessarily explore the number of treatment options available to each informant. Since the level of involvement of a patient will depend on the number of options available to her/him, we should have enquired about available information in more detail and considered it in the analysis.

Despite the above-mentioned limitations, we still believe that this study can offer several implications to increase patient satisfaction with regard to the decision-making process in the oncology setting in Japan. First and most importantly, we need to discuss the measures to more accurately understand each patient's preference regarding her/him roles in the decision-making process. Bruera and colleagues proposed that patient preferences should be assessed on a prospective basis by directly asking the patient rather than assuming that the existing level of communication will allow the physician to predict a strategy [35]. This approach toward prospectively and directly assessing patient preferences is important as evident from the results of a preceding study, which suggest that physicians, even those highly trained in communication skills, are not equipped to predict patient preferences regarding their role in decision-making [35]. Second, patients should be provided with (1) detailed information on the available treatment options and (2) sufficient time to deliberate on the options before being required to make a decision. Moreover, physicians should be aware that, merely attempting to involve patients into treatment-related decision-making without adequate information exchange and deliberation on the options can result in dissatisfaction on the part of the patients. Further, physicians should be advised on how to effectively utilize the available educational material to increase their patients' understanding of the disease and treatment options. Since around 2004, many evidence-based, clinical practice guidelines have been published in Japan, and some of the guidelines which originally targeted physicians have been re-edited with the help of patient groups and published for use by patients. In addition, we observed an increase in the number of reliable online cancer information services provided by the government, healthcare professionals, and patient groups [36]. These trends have facilitated information exchange and discussion between patients and physicians, because reliable cancer information services such as those provided by the American Cancer Society or National Cancer Institute in the US had long been lacking in Japan. The interviews for the current research were conducted in 2001, when these changes were still in their nascent stage. Patients who were more recently diagnosed might have been able to collect reliable information more efficiently than the informants of the current research were able to. Third, we need to inform the patients under the 'surrendering decision-making' category that they should express their preferences regarding their role in the decision-making process more explicitly. For this purpose, we also need to explore the reasons why such patients have a sense of resignation, in contrast to those who become more assertive in expressing their preferences after unsatisfactory experiences related to decision-making.

## Conclusion

This study revealed that (1) a marked variation exists in the Japanese cancer patients' preferences regarding their role in decision-making and (2) the main factor for patient satisfaction was respecting the patients' preferences regarding their role in decision-making. We need to create a better environment in hospitals and in society to facilitate patients in expressing their preferences and finding resources to support their decisions.

## Competing interests

The author(s) declare that they have no competing interests.

## Authors' contributions

YW was responsible for acquiring, analysing, and interpreting the data as well as making contributions to study conception and design and manuscript elaboration. MT participated in the analysis and interpretation of the data and made substantial contributions to manuscript conception and elaboration. IK made considerable contributions to manuscript conception and elaboration. All authors read and approved the final manuscript.

## Pre-publication history

The pre-publication history for this paper can be accessed here:


